# X-ray magnetic linear dichroism as a probe for non-collinear magnetic state in ferrimagnetic single layer exchange bias systems

**DOI:** 10.1038/s41598-019-54356-y

**Published:** 2019-12-03

**Authors:** Chen Luo, Hanjo Ryll, Christian H. Back, Florin Radu

**Affiliations:** 1 Helmholtz-Zentrum-Berlin für Materialen und Energie, Albert-Einstein-Strasse 15, 12489 Berlin, Germany; 2 Institute of Experimental and Applied Physics, University of Regensburg, 93053 Regensburg, Germany; 3 Institute of Experimental Physics of Functional Spin Systems, Technical University Munich, James-Franck-Str. 1, 85748 Garching b. München, Germany

**Keywords:** Magnetic properties and materials, Surfaces, interfaces and thin films

## Abstract

Ferrimagnetic alloys are extensively studied for their unique magnetic properties leading to possible applications in perpendicular magnetic recording, due to their deterministic ultrafast switching and heat assisted magnetic recording capabilities. On a prototype ferrimagnetic alloy we demonstrate fascinating properties that occur close to a critical temperature where the magnetization is vanishing, just as in an antiferromagnet. From the X-ray magnetic circular dichroism measurements, an anomalous ‘wing shape’ hysteresis loop is observed slightly above the compensation temperature. This bears the characteristics of an intrinsic exchange bias effect, referred to as *atomic exchange bias*. We further exploit the X-ray magnetic linear dichroism (XMLD) contrast for probing non-collinear states which allows us to discriminate between two main reversal mechanisms, namely perpendicular domain wall formation versus spin-flop transition. Ultimately, we analyze the elemental magnetic moments for the surface and the bulk parts, separately, which allows to identify in the phase diagram the temperature window where this effect takes place. Moreover, we suggests that this effect is a general phenomenon in ferrimagnetic thin films which may also contribute to the understanding of the mechanism behind the all optical switching effect.

## Introduction

Non-collinear magnetism is emerging as a crucially important trait of magnetic systems which are indispensable to antiferromagnetic spintronics^1^. Magnetic skyrmions, helical and conical states, canted spins specific to frustrated systems, domain walls in ferromagnetic and antiferromagnetic materials are all attempted to be controlled through external stimuli (electric currents, voltages, laser excitations, strain) towards functionalization for applications in modern devices. Moreover, non-collinear spin textures in ferrimagnets can give rise to anomalous Hall effect^2,3^, which enables readout in magnetic sensors. While important progress is made on the understanding of complex magnetic textures in single crystals, the miniaturization of devices requires nano-scaling of the materials, which leads to significant modifications of their bulk magnetic properties.

Among magnetic materials, rare-earth-transition-metals (RE-TM) ferrimagnetic alloys have attracted great interest because they exhibit superior flexibility in designing desired properties for ultimate functionality and as model systems for basic research in the field of spintronics. They can be easily engineered as two ferromagnetic oppositely oriented sub-lattices in form of thin films and nanostructures with controllable perpendicular anisotropy, variable net magnetization as a function of stoichiometry and tunable spin reorientation transition temperature^4^. They can also be assembled as heterostructures in form of spin valves and tunnel junctions^5,6^. For example, DyCo/Ta/FeGd has been demonstrated to exhibit interlayer exchange coupling and a tunable and robust perpendicular exchange bias at room temperature, which can be set without additional field cooling cycles^4,5^. The DyCo_5_ material has also been proposed to be suitable for heat-assisted magnetic recording near room temperature^7^. For the archetypical GdFeCo and other RE-TM ferrimagnets like TbFe, TbCo it has been demonstrated that their magnetization can be controlled using femtosecond laser pulses at large lateral length scales and at the nanoscale, without applying any external magnetic field^8–12^. RE-TM ferrimagnetic alloys can be tuned to behave as true antiferromagnets at a compensation temperature where the magnetic moments of the RE and TM sub-lattices are equal in size but oppositely oriented, leading to a zero net magnetization. For some RE elements with low orbital magnetic moments, comparable to the orbital magnetic moment of the TM element, the angular momentum may be quenched for a certain temperature which may lead to an acceleration of the precessional spin dynamics^13,14^. Ultrafast magnetization reversal across the compensation temperature of RE-TM alloys may deterministically provide the ultimate switching speeds that can be achieved today^15–19^.

The complex physics near the compensation temperature is enriched by one more fascinating effect. Anomalous magnetic behaviour in form of *wing shape* hysteresis loop has been reported in several RE-TM ferrimagnetic alloys that exhibit a perpendicular magnetic anisotropy such as, GdCo^20^, HoCo^21^, TbFe^22^, GdFe^23^, DyCo^24,25^, and GdFeCo^26–28^ thin films. When applying a magnetic field perpendicular to the sample, it is expected that the net magnetization will naturally align with the external field. However, a counter-intuitive effect is observed: the magnetization appears to diminish when the magnetic field overcomes a certain value, leading to an apparent decrease or even a vanishing magnetization of the sample.

Originally, this intriguing effect was interpreted based on models assuming an alloy composition gradient across the film thickness or even across lateral directions of the sample. According to these assumptions, a magnetization compensation temperatures will occur throughout the film, possible also due to local stoichiometry variations, and as a result the magnetic hysteresis loop will reflect the relative weight of the corresponding parts of the film^20,26^. These early models have been addressed critically in relation to similar observations in HoCo films^21^, questioning the original proposals based on the structural or magnetic inhomogeneities. Recently, the observation of similar anomalous loops in GdFeCo was observed to occur at faster time scales^23^. For this case the origin for the effect was suggested to be caused by a transient temperature range which extends over the compensation temperature of the film. Even more recently, similar anomalous magnetic behaviour in the same GdFeCo films was reported in equilibrium with the suggestion that its origin actually may relate to a spin-flop mechanism^28^. This last attempt to resolve the debate, however, comes at odds with previous observation of this effect in a DyCo_4_ film where it is suggested that the effect bears the characteristics of an exchange bias effect^24^. As a result, although this effect is of paramount importance for ultrafast magnetization research, its fundamental origin is still highly debated. We center our study on resolving the origin of the effect utilizing one of the most powerful modern experimental tools to magnetism, namely soft x-ray spectroscopy. Moreover, we suggest that this effect may explain the origin of all optical switching in ferrimagnetic films (see Discussion section and Supplementary).

## Results

We make use of x-ray circular magnetic dichroism (XMCD)^29^ and x-ray linear magnetic dichroism (XMLD) assembled from magnetic field dependent absorption spectra (XAS) measured by total electron yield (TEY) and by fluorescence yield (FY) to resolve the origin of the magnetic transition that occurs close to the compensation temperature of RE-TM ferrimagnetic alloys. XMCD is sensitive to the ensemble averaged orbital and spin contribution to the magnetic moments projected along the circular polarization direction which is parallel to the x-ray beam direction. Through the sign of XMCD one can distinguish the directional sense of the magnetic moments. However, through its magnitude one cannot uniquely discriminate on their eventual non-collinear arrangement with respect to magnetic domain formation. To achieve this capability, the XMLD contrast will be involved.

XMLD^30–34^ is the difference in XAS cross section for the $\overrightarrow{E}$ vector of linear polarized X-rays oriented parallel and perpendicular to the magnetic moments. XMLD depends on the square of the magnetic moment $\langle {M}^{2}\rangle$ and on the magneto-crystalline anisotropy, which makes it favorable for the study of antiferromagnetic systems. In spite of the key dependences to intrinsic magnetic properties, for 3d transition metal elements (Mn, Co, Fe) the size of the XMLD effect is extremely small, hindering its further development^32,35^. Also, it requires a rather high precision of reproducibility of energy set since the size of its sign changing contrast at the L_3_ resonant edge occurs in a narrow energy range. We exploit here, as demonstrated further below, the XMLD at the RE (Dy) edges which is expected to be much larger^30^ and occurs as a sizable intensity change at the M_5_ edge for both TEY and FY detection modes.

**Figure 1:**
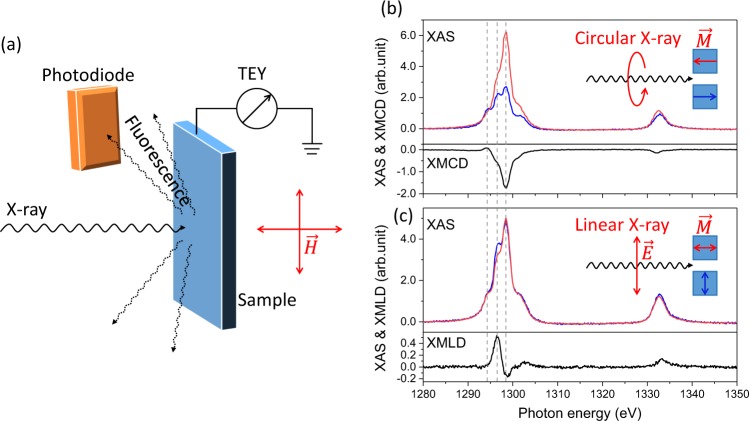
(**a**) Sketch of the XMCD and XMLD measurements, the fluorescence and TEY signals from the sample are recorded. (**b**) The XAS and XMCD spectra for the Dy M_4,5_ edges at 300 K. (**c**) The XAS and XMLD spectra for the Dy M_4,5_ edges at 300 K. The three peaks of Dy M_5_ edge are marked by the dashed lines at *E* = 1294.3, 1296.5 and 1298.4 eV. The data in panels (b,c) were both recorded in FY mode.

First we present the demonstration of XMCD and XMLD geometries and the novel characteristics of the XMLD effect at the Dy M edges through an experiment to probe the non-collinear states in DyCo_5_ ferrimagnetic thin films at room temperature. Figure 1(a) shows the experimental geometry for the surface and bulk measurements (see also Supplementary). All the measurements were performed in a perpendicular geometry, the bulk sensitive FY signal with a probing depth of ~100 nm^36^ was recorded by a photodiode located 2 cm away from the sample surface. Figure 1(b) shows the XAS and XMCD measurements for Dy at 300 K. The XMCD spectrum was obtained by taking the difference of (*σ*^+^ − *σ*^−^), where *σ*^+^ and *σ*^−^ represent the XAS spectra measured by FY and using the circular polarized X-rays with the magnetic field (*μ*_0_*H* = 1 Tesla) parallel and anti-parallel to the beam direction. The Dy XMLD spectrum was obtained by taking the difference of the XAS spectra measured by keeping the linear polarized X-rays $\overrightarrow{E}$ parallel to the synchrotron plane and perpendicular to the beam direction, and setting the direction of the magnetic moments perpendicular and parallel to $\overrightarrow{E}$, respectively, as shown in Fig. 1(c). The magnetic field of 1 Tesla was checked to saturate the magnetization of the sample whether in-plane or out-plane at room temperature. The field was applied parallel to the beam direction and perpendicular to it. As shown in the figure, there are three peaks at the Dy M_5_ edge, which are marked by the dashed lines. The maximum XMCD signal appears enhanced at the third peak whereas the maximum XMLD signal is located at the middle peak. The intensity difference at the M_5_ edge for the XMLD spectra is sufficiently large to be exploited for intensity measurements as a function of the external field. The sensitivity to the angular orientation between the magnetic moments and the direction of the polarization vector is clearly demonstrated: strong XMLD contrast appears by re-orienting the magnetic moments only, from parallel to perpendicular directions with respect to $\overrightarrow{E}$, using vectorial magnetic fields. To exclude further contributions to the XMLD contrast possible caused by crystalline electric field effect, further orthogonal field directions in plane of the sample were measured (Supplementary).

**Figure 2:**
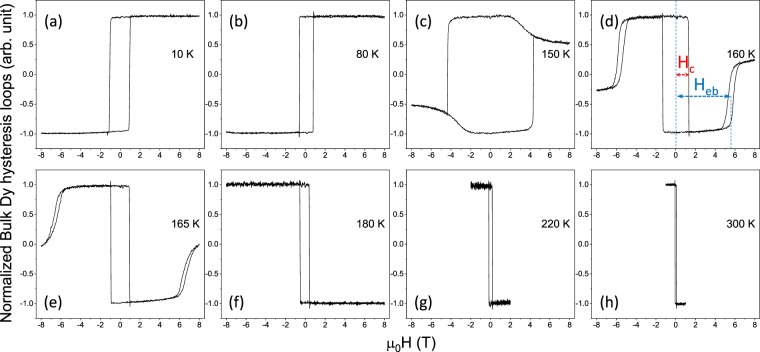
Temperature dependent hysteresis loops recorded by FY signal with circular polarized X-rays set to the Dy M_5_ edge (*E* = 1298.4 eV). The shift of the side hysteresis loop denoted as *H*_*eb*_ and the coercive field of the central loop denoted as *H*_*c*_ are depicted schematically in the panel (d).

To approach the compensation temperature, we performed temperature-dependent XMCD and hysteresis loop measurements at the Dy M_4,5_ and Co L_2,3_ edges. Part of the bulk sensitive hysteresis loops taken by measuring the FY intensity at the Dy M_5_ edge (*E* = 1298.4 eV) are shown in Fig. 2. The hysteresis loops signal reverses when the temperature crosses a critical temperature, called magnetization compensation temperature T_*comp*_. Also, the occurrence of the perpendicular magnetic anisotropy is clearly distinguished as the full remanent magnetization and by the sharpness of the magnetization reversal. Besides the main sharp reversal of the film, we observe an “anomalous” behaviour at higher fields which develops at temperatures higher than T_*comp*_, as shown in Fig. 2(d,e). This intriguing response of magnetization at higher magnetic fields is counter-intuitive in nature. For magnetic films which exhibits a net magnetization like ferromagnets, an external field will cause full magnetization, aligning the spins as the field is increased. By contrast, the “anomalous” hysteresis loops show that the sublattice magnetization decrease as the magnetic field is increased. This can be understood based on the fact the with soft x-rays we probe the individual sublattice magnetization which rotates away from the vertical direction. By contrast, the net magnetization, does indeed increases as expected, as a function of the external field (see the magnetic hysteresis loop shown in the Fig. S4 of the Supplementary File).

**Figure 3:**
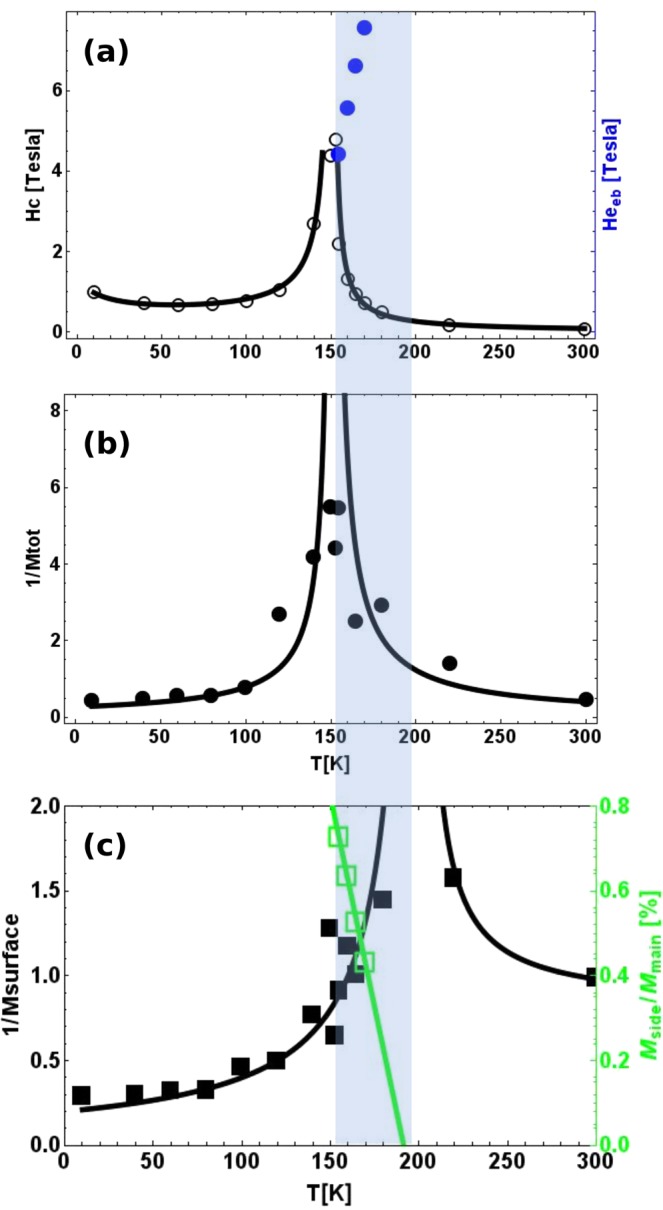
Phase diagram of the magnetic states as a function of temperature. (**a**) Temperature dependence of the coercivity field *μ*_0_*H*_*c*_ and the exchange bias field *H*_*eb*_. The values of *μ*_0_*H*_*c*_ were extracted from the rectangular hysteresis loops at the crossing point with respect to the magnetization axis. The shift of the side wings, denoted as *H*_*eb*_, were extracted at the half height of the side loops alone. The maximum value of *μ*_0_*H*_*c*_ is about 4.8 T slightly below T_*comp*_, whereas the highest measured shift side hysteresis loop is about 7 Tesla. (**b**) The inverse of the total net magnetic moment extracted by analyzing the XMCD spectra characteristic for the bulk part of the film (FY data). This show a divergent behaviour at the closely similar compensation temperature as in panel (a). (**c**) The inverse of the total net magnetic moment extracted by analyzing the XMCD spectra characteristic for the surface part of the film (TEY data). They also exhibit a divergent behaviour near 200 K, showing that the probed surface has a higher compensation temperature. As open square we plot the relative amplitude (height) of the side loops M_*side*_/M_*main*_, which is extracted from the hysteresis loops with side loops (see the Fig. S5(b) of the Supplementary). It shows a linearly decreasing behaviour with increasing temperature. A linear fit to these data points suggests the the side loop will cease to exist at a temperature around 192 K, which agrees well with the compensation temperature of surface part. In between these two compensation temperatures, the side hysteresis loops occur.

In Fig. 3(a) we plot the coercive field of the hysteresis loops and the shift of the side hysteresis loops as a function of temperature. The divergence of the coercive field, which occurs at the vanishing net magnetization of the film, reveals with good accuracy the absolute value of the intrinsic T_*comp*_ which is about 154 K, in close agreement with previous experiments and simulation results^37,38^. Also, we plot the field of the center of the wing hysteresis loops as a function of temperature, denoted as exchange bias field H_*eb*_. We observe that the shift of the side loop increases as the temperature increases up to the highest measured value of about 7 T. Due to the finite available external fields (up to 8 T) we could not further follow the shift of the side loop beyond the 7 T. This limitation is visible at 165 K, where the side loop begin to behave as a minor hysteresis loops. Instead, we can determine the limiting temperature where the side loop will vanish. To this end we show in Fig. 3(b,c) the inverse of the total net magnetic moments characteristic of bulk and surface, respectively. They are extracted from XMCD spectra measured by TEY and by FY (Supplementary). We observe that both the inverse of the bulk (filled circles) and the surface (filled squares) net magnetic moments exhibit a divergent behaviour, at 154 K for the bulk part of magnetization and at 200 K for the surface part. In addition we show in Fig. 3(c) (open square) the vertical opening of the side loop normalised to the vertical opening of the central hysteresis loop. This reflects the temperature evolution of the sublattice magnetization of the side loop projected to the direction of the magnetic field (see Supplementary, Fig. S5). This experimental observable decreases as the temperature increases suggesting that the vertical opening of the side loop cease to exist at higher temperatures. A linear fit of these data points intersects the abscissa at the compensation temperature of the surface part. The vanishing character of the side loop towards higher temperatures is observed also for the FeGd film (see Supplementary, Fig. S6).

As such, the system exhibits a different compensation temperature for the probed surface, which is about 50 K higher as compared to the bulk magnetization compensation^24^. In-between these two compensation temperatures the system is in a frustrated state. The occurrence of these two compensation temperatures correlates well with the temperature range where the anomalous magnetic behaviour occurs at high fields. Outside this region, we expect no side hysteresis loops because the surface and the bulk spins are in a stable configuration, basing on the relationship of the surface and bulk magnetic moments extracted from the XMCD spectra in Fig. 3(b,c). We mention that at 200 K which is closely above the compensation temperature of the surface, a hysteresis loop measured up to 14 Tesla (not shown) did not exhibit a side loop, but a rather saturated plateau.

**Figure 4:**
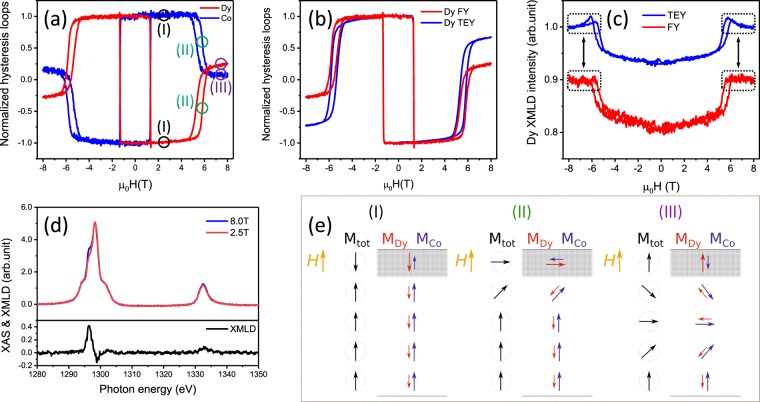
XMCD and XMLD measurements at 160 K. (**a**) Hysteresis loops measured by recording the FY signal with ***circular*** polarized X-rays at the third peak of the Dy M_5_ edges (*E* = 1298.4 eV) and the Co L_3_ edge (*E* = 777.0 eV). (**b**) Comparison of surface (TEY) and bulk (FY) hysteresis loops for Dy. (**c**) XMLD hysteresis loops measured by recording the FY and TEY signal with ***linear*** polarized X-rays at the second peak of the Dy M_5_ edges (*E* = 1296.5 eV). (**d**) XAS and XMLD spectra taken at 8 T and 2.5 T for Dy with linear polarized X-rays. (**e**) Sketch of the magnetic spin structures for the different states in panel (a).

To characterize the reversal of the anomalous loops we make use of the XMCD and XMLD effects, focusing on the relevant temperature of 160 K. Figure 4(a) shows the hysteresis loops at both Dy M_5_ edge and Co L_3_ edge. They exhibit a similar behaviour with opposite signs. This demonstrates that the magnetic moments of the Dy and Co sublattices are basically anti-parallelly coupled to each other, even when they enter the anomalous spin state at higher fields. At this stage, we can resolute that a spin-flop transition cannot be seen as the underlying mechanism for the drastic decrease of the sublattice magnetization. If such a spin-flop would appear in the Fig. 4(a), it would lead to a significant difference between the shape of the magnetization curves for Dy and Co, which is not observed here. In fact, they show a similar shape with opposite signs, indicating that they remain largely parallel across this transition. Similar results were observed further from the compensation temperature at 165 K, as shown in the Fig. S5(a) of the Supplementary. Due to the limit of the magnetic fields in our experiments, our results do not exclude the occurence of a spin-flop transition at very high fields. But for the region we explore, the mechanism of the anomalous loops is related to the exchange bias state that excites between compensation temperatures of bulk and the surface.

However, a weak non-collinear state between Co and Dy net magnetic moments can be distinguished by the difference of the relative magnetizations present at ±8 T. This observation is further supported by the demonstration of the same effect in a FeGd film (Supplementary). We can consider that the Co and Dy sublattices are essentially anti-parallelly oriented for all external magnetic fields. This, however, does not exclude a non-collinear behaviour for the elemental sublattices: comparing the surface and bulk hysteresis loops, see Fig. 4(b), clearly reveals that the surface signal is almost completely reversed at high fields while the bulk signal is only half reversed, which strongly indicates that the mechanism is due to the reversing of the surface magnetic moments.

By taking advantage of the strong linear dichroism of the Dy element at the M_5_ absorption edge, XMLD measurements were applied to understand the anomalous magnetic behaviour. Here, we use linear polarized X-rays to perform hysteresis loop measurements, as shown in Fig. 4(c). The XMLD hysteresis loops were recorded at the middle peak of the Dy M_5_ edges at *E* = 129.5 eV, where we observed a maximum XMLD signal in Figs. 1(c) and 4(d). From Fig. 4(c) one can see that there are two hysteresis-loop-like structures for both surface and bulk. The two loops appear at the same field of the XMCD ‘wing shape’ hysteresis loops and end up at the same level of intensity at ±8 T. These results reveal that parts of the magnetic moments rotate from the out-of-plane to the in-plane direction at high fields, which directly indicates the existence of non-collinear spin structure between the surface and the bulk. One needs to point out that there are also significant differences between the bulk and surface signals, which are located close to ±6 T in Fig. 4(c) (marked with dash rectangle boxes). As distinct from the bulk signal, the surface XMLD hysteresis loop reaches a maximum value around ±6 T, indicating that the surface magnetic moments rotate to the in-plane direction around this field, then it decreases and approaches flattening near ±8 T. Note that the same behaviour is observed also for the FeGd film shown in supplementary data (see Supplementary, Fig. S7(b)). This further indicates that the surface magnetic moments have a higher rotation angle at high fields. This is in full agreement with a simple domain wall structure initiated at the surface of the film.

Figure 4(d) shows the XAS spectra of Dy and their difference taken at 8 T and 2.5 T with linear polarized X-rays. By comparing the image with the standard XAS and XMLD measurements at 300 K, one can see that the XAS at 8 T is similar to the *σ*_||_ XAS while the XAS at 2.5 T is close to the *σ*_⊥_ XAS in Fig. 1(c). By defining the relative XMLD amplitude as (*σ*_||_ − *σ*_⊥_)/(*σ*_||_ + *σ*_⊥_), we get a value of ~4.4% at 160 K and ~5.6% at 300 K. Note that the XMLD amplitude is believed to be proportional to the square of the total magnetic moments *M*^2^ ^30^. The magnetic moments at 160 K are about ${m}_{bulk}^{160K}={m}_{s}+{m}_{l}=6.8{\mu }_{B}/{\rm{atom}}$ and ${m}_{surface}^{160K}=5.7{\mu }_{B}/{\rm{atom}}$, which are about 1.45 times of the magnetic moments at 300 K ${m}_{bulk}^{300K}=4.8{\mu }_{B}/{\rm{atom}}$ and ${m}_{surface}^{300K}=3.8{\mu }_{B}/{\rm{atom}}$. Thus the relative XMLD amplitude of 5.6% × 1.45^2^ = 11.6% can be expected at 160 K for the situation that all the magnetic moments align in-plane versus out-of-plane. Here the experimental value of 4.4% means that the in-plane contribution at 8 T is about $\sqrt{4.4 \% /11.6 \% }\approx 62 \%$ of the total magnetic moments. By assuming the magnetic moments are homogeneously oriented in the 180^0^ domain wall shown in Fig. 4(e-III), then one can estimate the domain wall thickness is about $20\,nm\times 62 \% \div({\int }_{0}^{\pi }\,\frac{\sin \,\theta }{\pi }d\theta )\approx 19\,nm$, where 20 nm is taken as the film thickness. An alternative approach is using a classical estimation for the domain wall thickness as $\pi \sqrt{A/K}$. If one assume that the exchange stiffness is about $A\approx 8\times {10}^{-8}\,erg/cm$ and the anisotropy constant is about $K\approx 9.84\times {10}^{4}\,erg/c{m}^{3}$ for our film also in agreement with Refs. ^24,39^, the domain wall thickness is about ≈28 nm. This suggests that the thin film can accommodate a partial domain wall.

## Discussion

Based on the experimental facts, one can draw a sketch of the spin structure for the anomalous magnetic behaviour, as shown in Fig. 4(e). The surface magnetic moments are always smaller than the bulk magnetic moments for both Dy and Co. At the magnetic remanence, the spins of Dy and Co are in a collinear state and anti-parallelly coupled to each other. Due to the strong exchange coupling between the surface and bulk, and due to the fact that the surface magnetism is dominated by Dy while the bulk magnetism is dominated by Co, the net moments of the surface would prefer to align anti-parallel towards the bulk moments. At high fields, this frustrated state becomes unstable, which forces the spin structure of the whole system to turn into a non-collinear state with an out-of-plane partial domain wall. Within this wall, the exchange energy is stored and released, which resembles closely exchange bias interactions with the difference that the atomic exchange is not affected by additional interfaces in an otherwise generic “two magnetic layers” system. By analyzing the net magnetic moments for the bulk and surface, separately, one can localize the temperature range where the occurrence of the side loop takes place. Within this temperature range the shift of the side loop increases as a function of temperature. This can be understood within the general theories for exchange bias^40^, which postulates the shift of the hysteresis loop is inverse proportional to the *M* × *t*, where M is the magnetization of the active magnetic layer and t is its thickness. In our case the active layer is the surface which has a compensation temperature of 200 K, therefore, when approaching this temperature the shift of the hysteresis should increase, as observed experimentally in Fig. 3.

A direct measure of the net magnetization is provided for two representative temperatures, at 155 K and 160 K in the section S3 of the Supplementary File. These data is measured by SQUID magnetometry and it fully supports the exchange bias character of the “wing-shaped” hysteresis loop through their pronounced asymmetric reversal shape which is an unique feature of the exchange bias phenomenon.

To suggest that this effect is a general phenomenon which occurs in thin ferrimagnetic films close to the compensation temperature, we provide supplementary data (Supplementary) on yet another system, namely FeGd ferrimagnetic film. There, the same effects are observed. Nevertheless, since FeGd has a lower magnetic anisotropy and stiffness (due to the nearly vanishing orbital moment of Gd), the temperature range where the shift of the hysteresis loop occurs is larger.

At a more general level, we speculate that our observations may have an impact towards deeper understanding of key aspects of ultrafast magnetic switching in ferrimagnetic films^8,17^. The mere occurrence of two compensation temperatures comprising two magnetic states in a frustrated arrangement should motivate further experiments which includes this peculiar phase diagram, also considering thickness^41^ as a control parameter. As an alternative mechanism for the all optical switching, measured from below compensation in an external field, we can assume an intersection between the intrinsic dynamical paths and the static ones. For instance, if the *H*_*eb*_ will scale down in the non-equilibrium state during the pump probe delay, one can transiently cross over the formation of the domain wall state leading to the so called transient-ferromagnetic state observed for an FeCoGd film in^17^. In fact, strong evidence for this scenario is seen in the Fig. S7(a) (Supplementary). There, we observe that the regions between the −6 T and −2 and between 2 T and 6 T are very similar to the so called transient ferromagnetic-like state observed in all optical switching experiments on FeCoGd samples. The projection of the Gd magnetization on the field direction is opposite (after a crossing field equal to 3.5 T) with respect to the magnetization at magnetic remanence, whereas the projection of Fe sublattice magnetization is vanishing. As such, the total net magnetic moment of the whole film is oppositely oriented at high fields (larger than 3.5 T) with respect to the net magnetic moment in lower applied fields (smaller then 3.5 T). Thus, assuming that during the pump probe delay, the time-dependent non-equilibrium states causes the system to cross the critical field for the formation of this “spring” spin configuration, one can understand the origin of the all optical switching in ferrimagnetic thin films by analogy to the effect we reported here. Note, that this mechanism excludes the occurrence of the same all optical switching effect in ferromagnetic materials, because this magnetic spring does not occur in these materials. Instead, the thermally assisted all-optical switching may (disjunctively from the effect we report) take place in ferromagnetic as well as in ferrimagnetic materials as considered in Refs. ^42,43^.

In conclusion, DyCo_5_ ferrimagnetic thin films were investigated with XMLD and XMCD techniques. An anomalous ‘wing shape’ hysteresis loop, referred to as atomic exchange bias effect with a large exchange bias field of *μ*_0_*H*_*EB*_ up to a maximum measurable value of 7 Tesla, was observed slightly above the compensation temperature T_*comp*_ ≈ 154 K. The origin of this effect which is demonstrated to be mediated by the formation of an out-of-plane partial domain wall during the hysteresis measurements, is directly confirmed via XMLD measurements. Such a huge perpendicular exchange bias effect in a single film may be a good candidate for future perpendicular magnetic recording applications. The technique of using the XMLD contrast at the rare earth M_4,5_ edges to probe the non-collinear states could be very useful for characterization of non-collinear magnetism which is intimately related to the spintronics research field.

## Methods

### Sample preparation

The 20 nm thick DyCo_5_ thin films were grown on sapphire substrates by magnetron sputtering (MAGSSY chamber at HZB) in an ultra-clean Argon atmosphere of 1.5 × 10^−3^ mbar with a base pressure of <2 × 10^−8^ mbar at room temperature. The stoichiometry of the ferrimagnetic alloy was controlled by varying the deposition rate of Co and Dy targets in a co-evaporation scheme. A 3 nm Ta capping layer was grown on top of the samples to prevent surface oxidation. We have characterized the lateral homogeneity using energy dispersive scanning X-Ray spectroscopy technique (EDS), but were unable to observe any phase separation at the sensitivity level of about few hundred nanometers provided by this method.

### X-ray absorption spectroscopy

The XAS measurements were performed at the VEKMAG end-station^44^ installed at the PM2 beamline of the synchrotron facility BESSY II. This end station offers unique capabilities for this type of research, since it provides a vector magnetic field option with a maximum magnetic field up to 9 Tesla in the beam direction, 2 Tesla in the horizontal plane and 1 Tesla in all directions for a temperature range of 2 K–500 K. The XAS spectra were recorded by means of total electron yield (TEY) and by fluorescence yield (FY), and for each constituent element, separately.

The TEY is measured by recording the drain current as a function of the x-ray photon energy normalized by a Ta grid x-ray monitor mounted in a magnetically shielded environment as the last optical element before the sample. The TEY is known to be surface sensitive, providing information over the escape length of the electrons which exhibits a mean free path of about 3 nm. As such, the surface magnetic properties are provided in a selective manner by this recording channel.

The FY is measured by a magnetically insensitive x-ray detector, placed at 2 cm away from the sample surface. FY is a photon-in photon-out spectroscopic technique which provides information integrated over the penetration depth of the x-ray, which can be of order of tens of nm. The depth sensitivity depends on the photon energy, absorption cross-section, and on the stoichiometry of the film. As such, the FY provides magnetic information for the whole film thickness, which we denote as “bulk” sensitive. The spectra are recorded as function of the x-ray energy and normalized by the same magnetically shielded x-ray monitor.

## Supplementary information

Supplementary Information
