# Supplementary material for: X-ray magnetic linear dichroism as a probe for non-collinear magnetic state in ferrimagnetic single layer exchange bias systems
Source: Sci Rep. 2019 Dec 3;9:18169. doi: 10.1038/s41598-019-54356-y (PMC6890699; doi:10.1038/s41598-019-54356-y)
Supplement: Supplementary file 1 — Supplementary Information [file 41598_2019_54356_MOESM1_ESM.pdf]

Supplementary information for:

X-ray magnetic linear dichroism as a probe for non-collinear magnetic state in ferrimagnetic single layer exchange bias systems

Chen Luo[1,2,3,*], Hanjo Ryll[1], Christian H. Back[2,3], and Florin Radu[1,]**

[1]Helmholtz-Zentrum-Berlin für Materialen und Energie, Albert-Einstein-Strasse 15, 12489 Berlin, Germany

[2]Institute of Experimental and Applied Physics, University of Regensburg, 93053 Regensburg, Germany

[3]Institute of Experimental Physics of Functional Spin Systems, Technical University Munich, James-Franck-Str. 1, 85748 Garching b. München, Germany

[*] *chen.luo@ur.de*

[**] *florin.radu@helmholtz-berlin.de*

October 1, 2019

Supplementary Notes

S1 XMLD: experimental geometries utilizing vectorial magnetic fields

We describe below the experimental geometries for the linear dichroism measurements using vector magnetic fields on the $Ta/DyCo_5/Al_2O_3$ sample of the main body of the paper. This demonstrates in a self-consistent manner the sensitivity of the XMLD contrast to the angle of magnetization direction with respect to the direction of the linear polarization. There are two common ways to measure a XMLD spectra. One is to keep the magnetization along the easy direction and to rotate the polarization direction, the other one is to keep the linear polarization direction fixed and change the magnetization direction [1]. Here, by taking advantage of the 3D vector magnet of the VEKMAG end-station, the second method is being used for our measurements. We set the linear polarization $\vec{E}$ oriented perpendicular to the beam direction and parallel to the storage ring plane. The sample is oriented perpendicular to the beam direction, as for transmission geometry. The magnetization of the sample is set oriented by the external field in three orthogonal directions: $H_{IP}^{\parallel}$, $H_{IP}^{\perp}$, and $H_{OP}^{\perp}$, as shown in Fig. S1(a). The OP and IP indexes refer to the direction of the magnetization with respect to the sample, namely out-of-plane and in-plane, respectively. The $\parallel$ and $\perp$ indexes refer to the orientation of the magnetization with respect to the linear polarization direction of the X-rays, namely parallel or perpendicular to $\vec{E}$, respectively.

The XAS spectra and their difference are shown in Fig. S1(b,c,d). The difference between the spectra measured for the orthogonal directions $H_{IP}^{\parallel}$ and $H_{OP}^{\perp}$, as well as for $H_{IP}^{\parallel}$ and $H_{IP}^{\perp}$, do show a XMLD contrast (black line in Fig. S1(c,d)). By contrast, when we compare the spectra recorded for $H_{IP}^{\perp}$ and $H_{OP}^{\perp}$, we observe a vanishing XMLD contrast (black line in Fig. S1(b)). This demonstrates that the XMLD contrast is sensitive only to the orientation between the direction of magnetization with respect to the direction of the linear polarization. Given that the amplitude of the XMLD is large, reaching a $\sim 5.6\%$ at room temperature, the intensity difference at the middle peak of the M_5 edge allows for detecting non-collinearity between the magnetization and the direction of the external field during hysteresis measurements, as described in the main body of the paper.

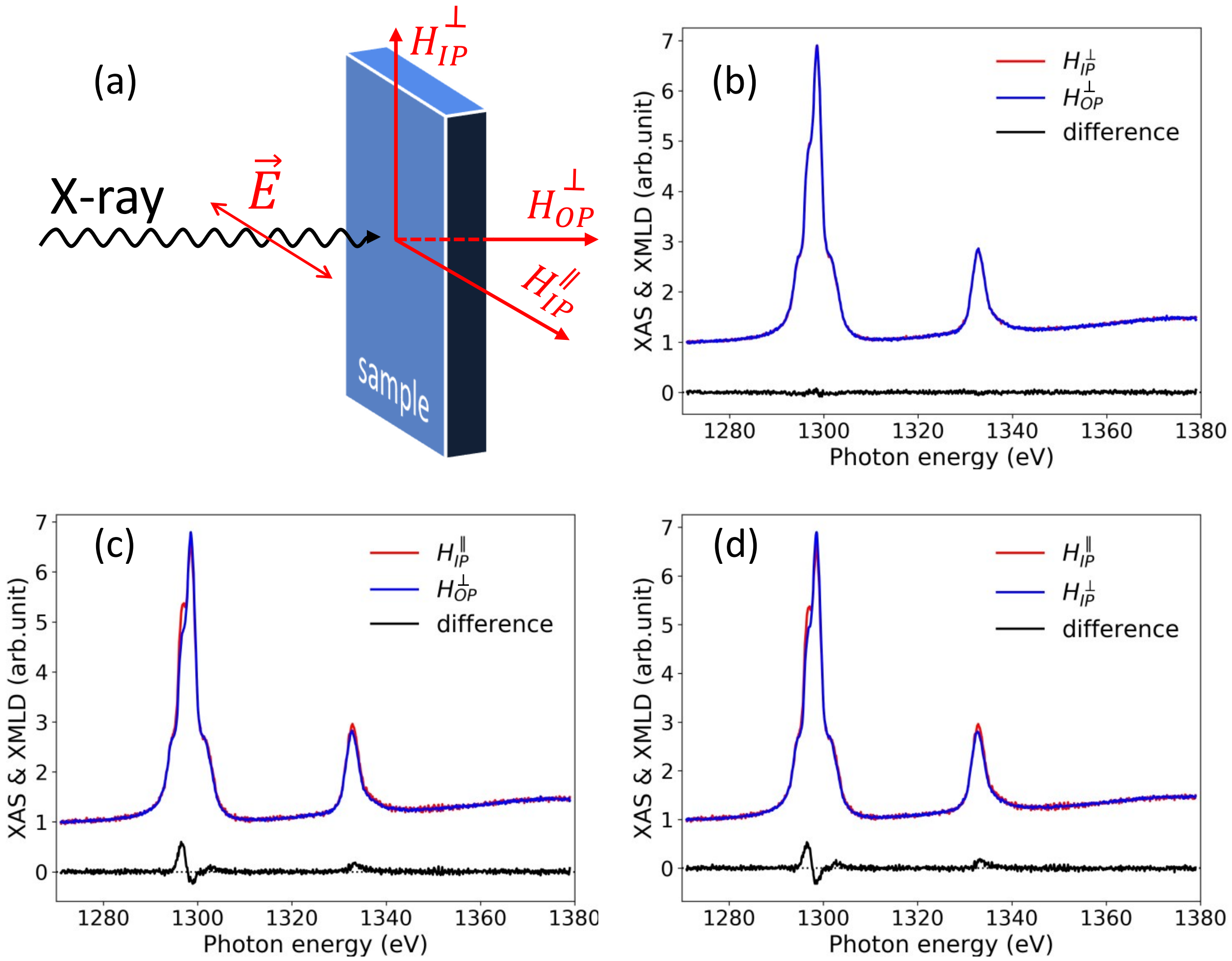

Figure S1: (a) Sketch of the XMLD measurements at room temperature. Here $H_{IP}^{\parallel}$ represents the field direction which lies in-plane and parallel to the $\vec{E}$ vector of the linear polarized X-rays, $H_{IP}^{\perp}$ represents the field direction which lies in-plane and perpendicular to $\vec{E}$, and $H_{OP}^{\perp}$ is the out-of-plane direction (perpendicular to the $\vec{E}$). (b,c,d) show the XAS spectra measured at $H_{IP}^{\parallel}$, $H_{IP}^{\perp}$ and $H_{OP}^{\perp}$, as well as their differences. The XAS spectra were measured by recording the FY as a function of x-ray energy across the M_5 and M_4 edges of Dy.

S2 XMCD: analysis of the magnetic moments

Due to the existence of the self-absorption and radiative decay effects, the FY spectra may exhibit a nonlinear dependence with respect to the absorption cross section [2, 3]. To compare the difference between the XMCD measured in FY, TEY and transmission mode, we prepared a calibration sample Ta(2.5 nm)/$DyCo_5$(20 nm)/Ta(5 nm) grown on a 150 nm thick Si_3N_4 membrane substrate. This allows us to measure the XAS and XMCD using TEY, fluorescence and transmission signals at the same time, as shown in Figure S2 (a,b,c). For a direct comparison, we have re-plotted all three XMCD spectra in Figure S2 (d). Comparing the XMCD spectra measured in transmission with the XMCD spectra measured in TEY mode, we observe that they have an identical line shape, with a clear difference in amplitude. This directly demonstrates that the magnetic moments of the surface are smaller as compared to the magnetic moments of the bulk part. However, the magnetic moments of the bulk measured by transmission and FY should have the same amplitude, which should be reflected in a similar amplitude and line-shape for the XMCD spectra. This is, however, not the case as directly demonstrated in Figure S2 (d): the amplitude of the XMCD spectra is similar for the transmission and FY modes, but the XMCD line-shape measured by FY exhibits an enhanced spectral feature at 1294.3 eV. This difference prevents an accurate determination of the spin and orbital moments through FY measurements. Nevertheless, we have applied the sum rules to the FY and transmission XMCD spectra and obtained the following magnetic moments at room temperature: $m_{total}^{FY} = 4.8\mu_B$/atom and $m_{total}^{TR} = 3.9\mu_B$/atom. One can see that the magnetic moment obtained from the fluorescence spectra is about 23% larger as compared to the value from the transmission spectra.

We then further use this scaling factor to correct for the magnetic moments extracted by applying the sum rules to the temperature dependence of the Dy XMCD spectra measured by FY in the main paper. For XMCD measured by FY for Co (not shown), this factor turned out to close to 1. We observe that both Co and Dy exhibit lower moments on the surface for the whole temperature range, with different percentages: the Co surface has about 67% of the bulk net moment value, whereas the Dy surface has about 84% of its bulk value. The fact that the Co moment is reduced stronger as compare to the Dy moment may indicate a non-collinear arrangement between the Co and the Dy spins at the top surface. This can be a result of a reduced surface coordination. The results are plotted in Fig. S3 to serve as the basis for the observed difference between the compensation temperatures of bulk and surface parts of the film.

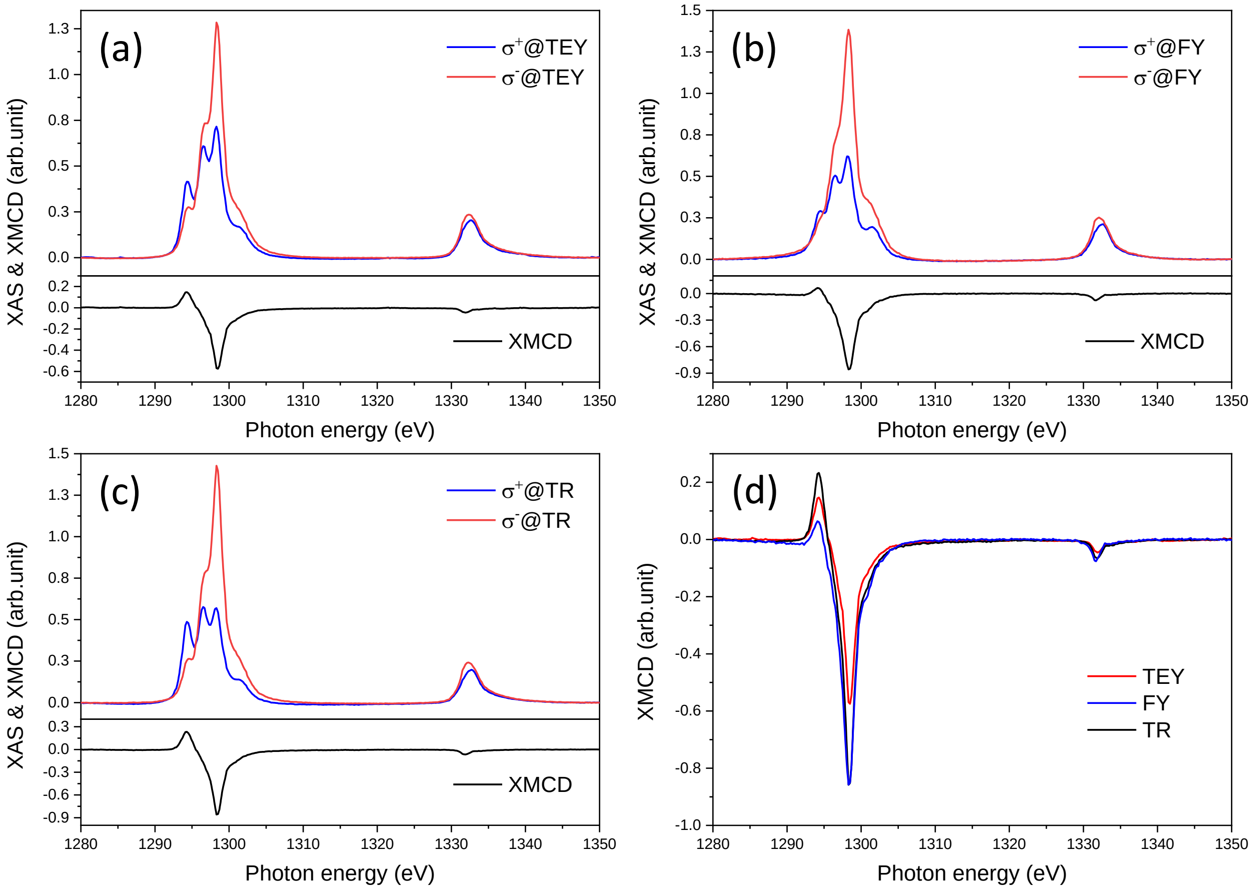

Figure S2: The Dy XAS and XMCD spectra measured by recording the TEY (a), FY (b) and transmission signals (c). (d) Comparison between the TEY, FY and transmission XMCD spectra.

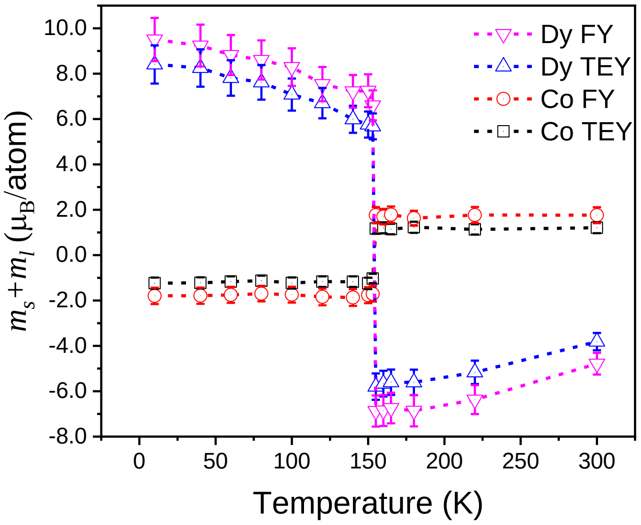

Figure S3: Temperature dependence of the total magnetic moments $m_s + m_l$. The spin m_s and orbital m_l magnetic moments were obtained by applying the XMCD sum rules [4, 5, 6] for the XAS and XMCD spectra measured at remanent magnetization. The sign of the magnetic moments reverses after crossing T_{comp}.

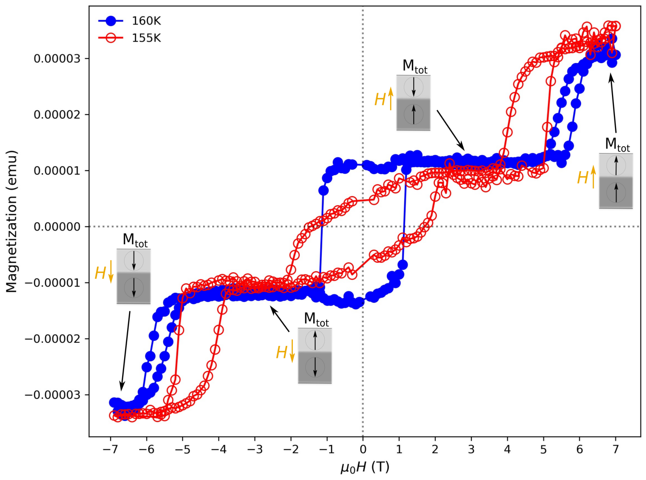

Figure S4: The out-of-plane M-H curves measured by SQUID at 155 K (red open circles) and 160 K (blue filled circles) after subtracting the background signal of sample holder and of the substrate. The insets are the sketch of the net moment structures based on the two-magnetic-layer model.

S3 SQUID measurements of the net magntization of the DyCo5 film

The temperature dependent of the net magnetization are performed by a SQUID magnetometer (Quantum Design) which provides a maximum magnetic field of 7 Tesla. The original sample was cut in small pieces and measured. After subtracting the background signal, the 'wing shape' hysteresis loops at 155 K and 160 K are shown in Fig. S4. Although the total net magnetization is very weak ($\sim 10^{-5}$ emu) due to the temperature proximity to the magnetization compensation temperature, a magnetic hysteresis loop could be measured. The hysteresis loop exhibits a central part and the "wing-shaped" hysteresis loops. The central part does reflects the sum of the net magnetization of the surface and of the bulk part projected along the applied magnetic field. The side-loops are shifted with respect to the external field axis and display a pronounced asymmetric reversal. The shift of the side loops agrees well with as the element-specific hysteresis loop presented in the main body of the manuscript.

Note that the net magnetization appears to saturate at high magnetic field, whereas the element specific data measured by XMCD do not exhibit a full saturation for the wing-shaped parts. These differences between SQUID and XMCD are consistent with the domain wall formation, since the XMCD data provides the averaged sublattice magnetization which cannot reach saturation as a domain wall. As a result they agree well with each other within the two-magnetic-layer model discussed in the main paper. Moreover, comparing the hysteresis loops for the two temperatures, we observe that the magnetization change of the central loop increases while the magnetization change of the side loop decreases with the increasing temperature. This behaviour agrees well with the assumption that the surface have another compensation temperature which is higher than the bulk, because the bulk magnetization gets larger when the temperature is higher the T_{comp}^{bulk} while the surface magnetization gets smaller when the temperature is closer to the $T_{comp}^{surface}$.

Note also, that this data suggests that the ground state at a temperature which lies between the magnetization compensation temperatures of the bulk and surface exhibits an antiparallel state (see cartoons sketches of the relative layer orientations which are inserted in Fig. S4). The net magnetization of the top layer is directed downwards with respect to the field axis, whereas the bottom (bulk) net magnetization part is directed upwards, when lowering the external field from positive towards negative values. The coupling between the two net magnetizations is given by the direct exchange of the elemental sublattices which are directly probed with the element-specific XMCD and XMLD techniques. As a result this is a strong coupling. For instance, theoretical considerations suggests that more the 100 T is necessary to break this coupling strength. As such,

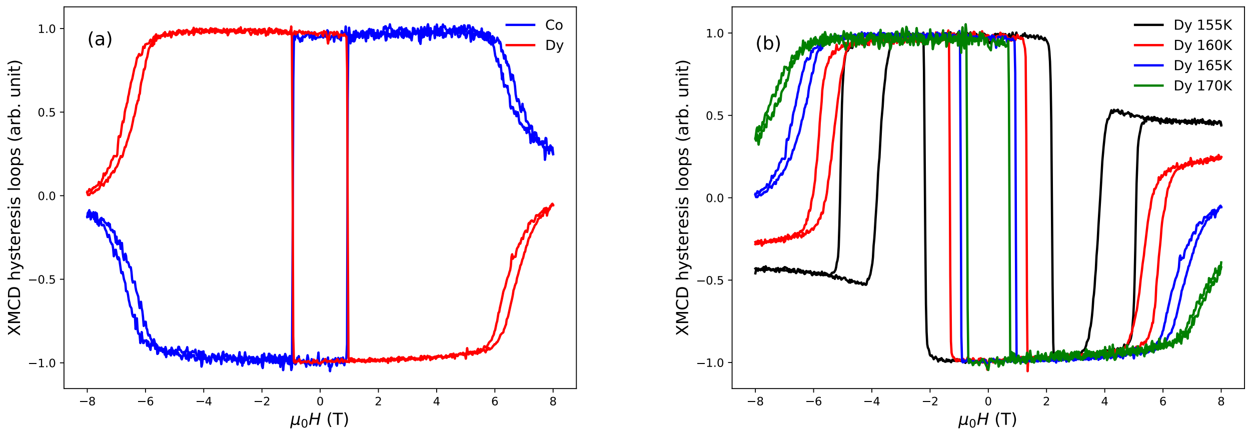

Figure S5: (a) The element-specific hysteresis loops at 165 K. From the results measured further from the compensation temperature, one can see that the effect is very similar to 160K, the only differences are the H_{eb} increased and the size of shape of the hysteresis loops is slightly reduced. The antiparallel orientation of the sublattice magnetizations is clearly seen by comparing the red (Dy) and blue curves (Co). (b) The comparison of the element-specific hysteresis loops with side loops from 155 K to 170 K. It clearly shows the H_{eb} increases with increasing temperature, meanwhile the 'size' of the side loops decreases with the increasing temperature, indicating that the side loops will disappear above a certain temperature (which is about 192 K, see Fig. 3(c) in the main text).

the ground state is similar to an exchange bias state of two layer coupled antiferromagnetically. When applying a positive magnetic field, the top layer will sense a force which will try to rotate it towards the field direction. The bottom layer is already parallel to the field direction, therefore, the higher the external field the harder it will be hold oriented upwards. As a result, this configuration behaves as a hard/soft exchange bias bilayer. The formation of the domain wall, stores the otherwise enormous coupling energy accommodating the saturated state, namely top and bottom magnetization pointing nearly parallel to the magnetic field.

S4 Temperature dependence of the sublattice magnetization of the DyCo5 film

We show in Figure S5(b) the sublatice magnetization of the Dy and Co measured by FY at 165K. This temperature is closer to the compensation temperature of the surface, complementing the Figure 4(a) (main manuscript body) which shows data measured at a temperature closer to the compensation temperature of the bulk part. We notice that the sublattices magnetization remain antiparallel oriented also at this temperature, demonstrating that this effect is characteristic for the whole temperature range where the side loop occurs.

In Figure S5(b) the temperature dependence for the Dy sublattice magnetization is shown fore more temperatures, namely for 155K, 160K, 165K and 170K. The measurements were performed by FY at the M5 edge of Dy and within a temperature range bordered by the compensation temperature of the bulk and surface.We notice that side loops exhibit a horizontal shift with respect to the field axis , but also a change of the vertical opening of the loop. This magnetization of the side loop reaches a maximum close to the compensation temperature of the bulk part and decreases as the temperature approaches the compensation temperature of the surface part. This suggest that at a certain temperature the side hysteresis loop will cease to exist. The sublattice magnetization of the side loop normalised to the magnetization of the central hysteresis loop is plotted in the Figure 3(c) in the manuscript body as open squares. A linear fit of this experimental

observable intersects the abscissa to the surface compensation temperature.

S5 Support study of the atomic exchange bias in FeGd film

For the purpose of supporting our results and method, and to demonstrate a general character of our observations, we show one more model system, namely a FeGd ferrimagnetic thin film. The sample has the following structure Ta(2.5 nm)/$Fe_{77}Gd_{23}$ (20 nm)/Ta(5 nm)/Si_3N_4. The Gd layer exhibits a nearly vanishing orbital magnetic moment, therefore the magnetic anisotropy and the stiffness of the film are much lower as compared to the Dy based alloys. As such it can be used as soft magnetic element in ferrimagnetic spin valves and it is the preferred system for the all optical ultrafast magnetic switching effect.

In Figure S6(a) we show the dependence of the coercive field and the shift of the side loop as a function of temperature. The coercive field exhibits a typical divergent behaviour at the compensation temperature which for this sample is about 25 K. Above the compensation temperature, the system develops an additional hysteresis loop denoted as H_{eb} which increases steadily up to a maximum measured value of about 6.2 T. Comparing this phase diagram with the one of $DyCo_5$ film we notice a similar character, but with some markedly differences: the temperature range where the atomic exchange bias occur is larger for FeGd (more 100 K) as compared to $DyCo_5$. Also, the temperature dependence of the H_{eb} shows deviations from a linear behaviour which is actually expected within the theories of exchange bias effect. By applying the sum rules, the surface and bulk magnetic moments for Fe and Gd are extracted from the TEY and transmission XMCD spectra. The inverse of the total net magnetic moments characteristic of bulk and surface are shown in Fig. S6(b,c), respectively. We observe that both the inverse of the bulk and the surface net magnetic moments exhibit a divergent behaviour, which is similar to the $DyCo_5$ sample in the main manuscript. In Fig. S6(c) we show the amplitude of the side loop (which corresponds to the magnetization projection along the field direction) devided by the amplitude of the central hysteresis loop (which corresponds to the magnetization of the main main hysteresis loop) as open squares. This experimental observable exhibits a linear character demonstrating the the amplitude of the side loop diminishes as the temperature increases. A linear fit of theses data points intersects the abscissa at the compensation temperature of the surface part, suggesting the the vertical opening of the side loop cease to exist outside the frustrated region. These results supports very well our finding that the effect only exists between the two compensation temperatures of bulk and surface.

In Figure S7 we provide an overview of the magnetic behaviour for a constant temperature equal to 35 K. The sample has been measured in transmission and TEY modes. The element specific (for Fe L_3 and Gd M_5 edges) magnetic hysteresis loops (Figure S7(a)) were measured in transmission mode (bulk part) with circular polarized beam (XMCD) impinging perpendicular to the sample surface. They display a central rectangular loop which is characteristic for the perpendicular anisotropy, and the side loops. These hysteresis loops clearly show that the Fe and Gd sub-lattice magnetizations are anti-parallelly oriented with respect to one another. At the highest fields, one notices that a weak non-collinearity occurs. Strikingly, the regions between the -6 T and -2 and between 2 T and 6 T are very similar to the so called transient ferromagnetic-like state observed in all optical switching experiments on FeCoGd samples [7] (see also the Discussion section in the main manuscript).

The hysteresis loops with linear polarization (XMLD) for both the surface (TEY) and the bulk (transmission) parts exhibit a clear imprint of a large rotation of the sub-latices from out-of-plane direction to an in-plane direction (Figure S7(b)). The differences between the surface and the bulk are also clearly observed, suggesting that the surface spins are more susceptible to the external fields. Finally, the XMLD spectra are shown for both TEY and transmission modes in Figure S7(c, d). Similar to Dy, the Gd does also exhibit a large XMLD contrast which can be used for non-collinearity studies.

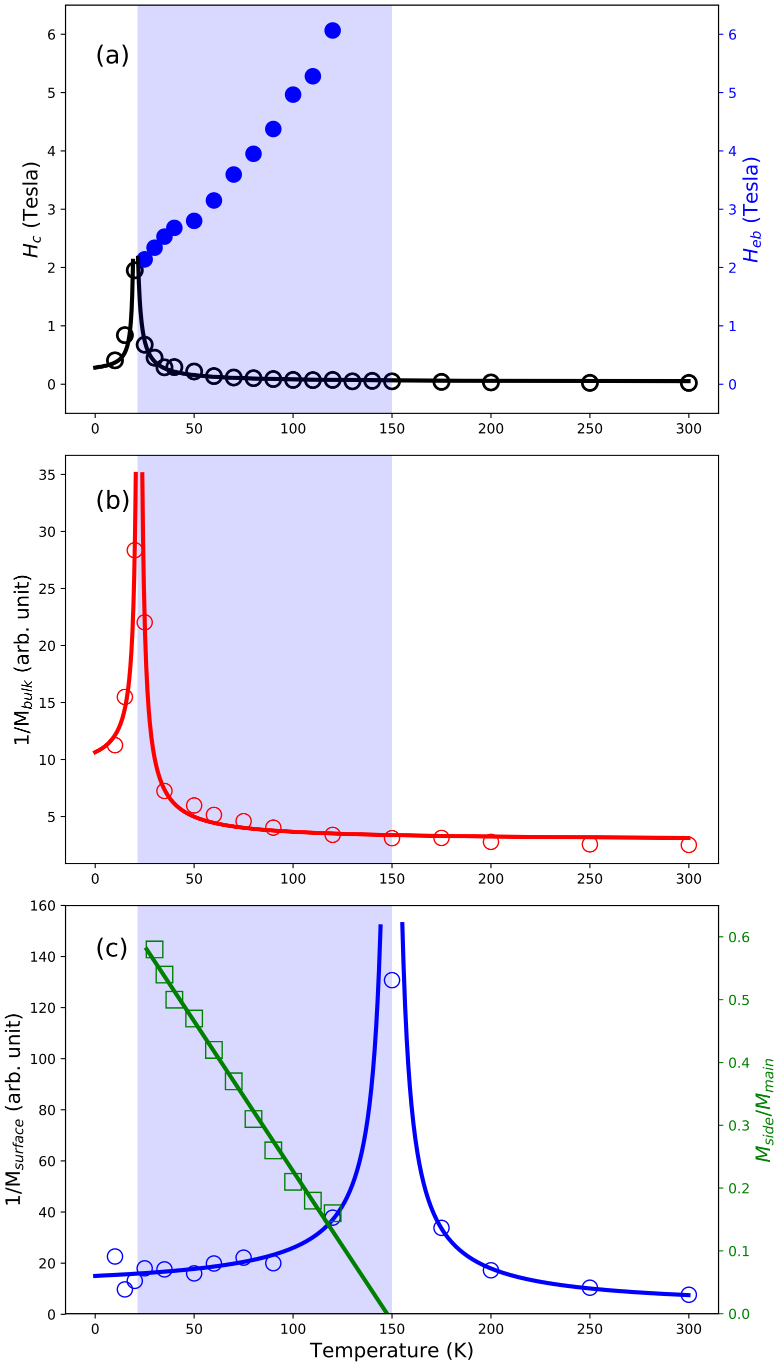

Figure S6: (a) Temperature dependence of the coercive field H_c (open black circles) and the exchange bias field H_{eb} (filled blue circles) for an FeGd film. The values of H_c were extracted from the rectangular hysteresis loops at the crossing point with respect to the magnetization axis. The shift of the side wings, denoted as H_{eb}, were extracted at the half height of the side loops alone. The maximum value of H_c is about 2 T slightly aside T_{comp}, whereas the highest measured shift of the side hysteresis loop is about 6.2 Tesla. (b) The inverse of the total net magnetic moment extracted by analyzing the XMCD spectra characteristic for the bulk part of the film (transmission data). This show a divergent behaviour at the closely similar compensation temperature as in panel (a). (c) The inverse of the total net magnetic moment (open blue circles) extracted by analyzing the XMCD spectra characteristic for the surface part of the film (TEY data). They also exhibit a divergent behaviour near 150 K, showing the probed surface has a higher compensation temperature. Meanwhile, the relative amplitude (height) of the side loops M_{side}/M_{main} (open green squares), which extracted from the hysteresis loops, shows a linearly decreasing behaviour with increasing temperature. A linear fit demonstrates that the side loops magnetization disappears at around 150 K, which agrees well with the compensation temperature of the surface part. In between these two compensation temperatures, the side hysteresis loops occur. The lines are guides to the eyes.

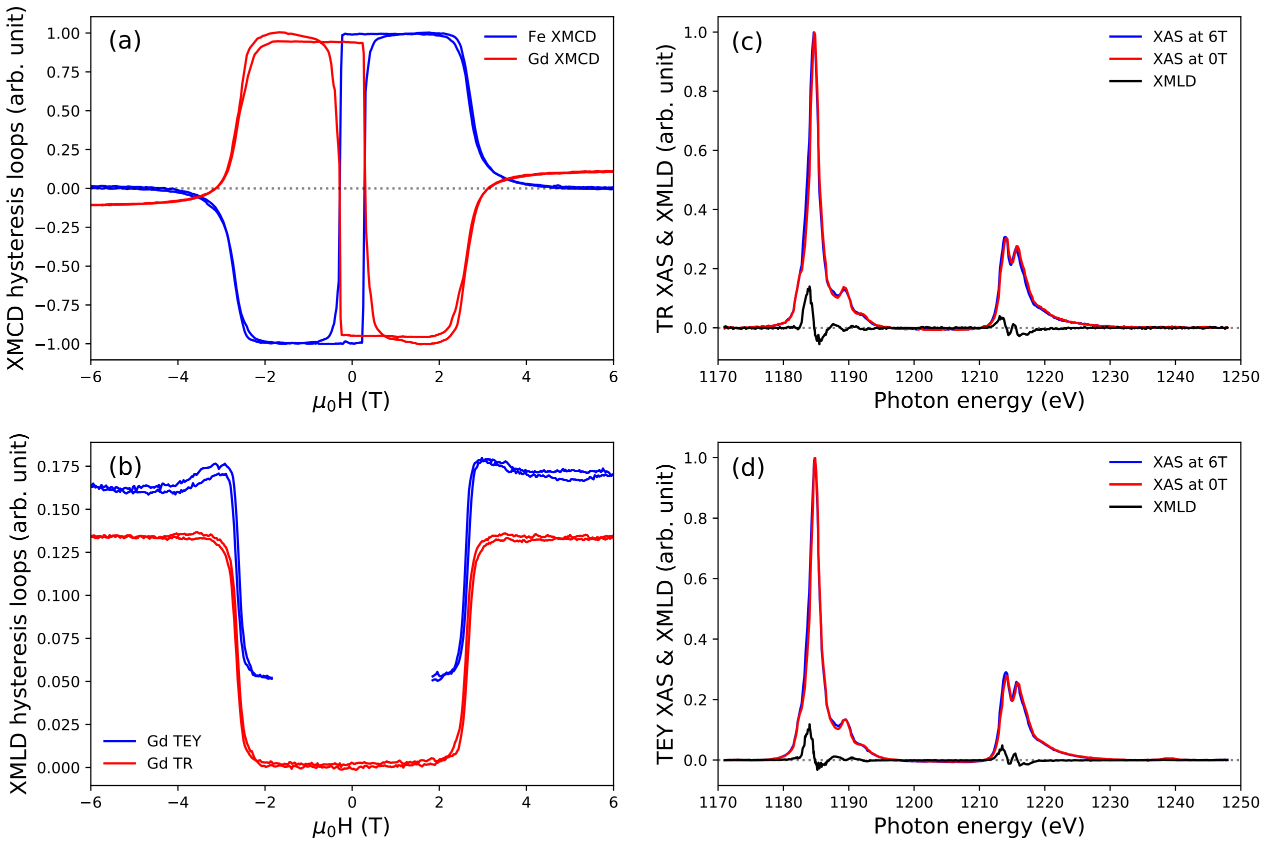

Figure S7: (a) The out-of-plane XMCD hysteresis loops are measured by recording the transmission signals with ***circular*** polarized X-rays at the Gd M_5 edge $E = 1184.9$ eV and at Fe L_3 edge $E = 707$ eV. They clearly demonstrate that the magnetic moments of the Gd and Fe are coupled anti-parallel one to each other. (b) The XMLD hysteresis loops are measured using the ***linear*** polarized X-rays at the Gd M_5 edge $E = 1183.7$ eV. Note that, for the TEY loop the data at smaller fields are not shown due to a common artefact caused by the electrons near zero field during field sweep measurements, leading to large distortions of the curve. (c,d) The XAS and XMLD spectra measured at 0T and 6T. Here we observe a $\approx 13.5\%$ XMLD signal for transmission (c) and $\approx 10.6\%$ for TEY (d).

References

[1] Gerrit van der Laan. Magnetic linear x-ray dichroism as a probe of the magnetocrystalline anisotropy. *Phys. Rev. Lett.*, 82:640–643, Jan 1999.

[2] S. Eisebitt, T. Böske, J.-E. Rubensson, and W. Eberhardt. Determination of absorption coefficients for concentrated samples by fluorescence detection. *Phys. Rev. B*, 47:14103–14109, Jun 1993.

[3] M. Pompa, A. M. Flank, P. Lagarde, J. C. Rife, I. Stekhin, M. Nakazawa, H. Ogasawara, and A. Kotani. Experimental and theoretical comparison between absorption, total electron yield, and fluorescence spectra of rare-earth M_5 edges. *Phys. Rev. B*, 56:2267–2272, Jul 1997.

[4] Paolo Carra, B. T. Thole, Massimo Altarelli, and Xindong Wang. X-ray circular dichroism and local magnetic fields. *Phys. Rev. Lett.*, 70:694–697, Feb 1993.

[5] B. T. Thole, P. Carra, F. Sette, and G. van der Laan. X-ray circular dichroism as a probe of orbital magnetization. *Phys. Rev. Lett.*, 68:1943–1946, Mar 1992.

[6] C. T. Chen, Y. U. Idzerda, H.-J. Lin, N. V. Smith, G. Meigs, E. Chaban, G. H. Ho, E. Pellegrin, and F. Sette. Experimental confirmation of the x-ray magnetic circular dichroism sum rules for iron and cobalt. *Phys. Rev. Lett.*, 75:152–155, Jul 1995.

[7] I Radu, K Vahaplar, C Stamm, T Kachel, N Pontius, HA Dürr, TA Ostler, J Barker, RFL Evans, RW Chantrell, et al. Transient ferromagnetic-like state mediating ultrafast reversal of antiferromagnetically coupled spins. *Nature*, 472(7342):205–208, 2011.
